# Percutaneous Emergency Needle Caecostomy for Prevention of Caecal Perforation

**DOI:** 10.1155/2017/1090769

**Published:** 2017-08-15

**Authors:** Alexandra M. Limmer, Zackariah Clement

**Affiliations:** Department of Surgery, Liverpool Hospital, Liverpool, NSW 2170, Australia

## Abstract

Caecal perforation is a life-threatening complication of large bowel obstruction with a reported mortality of 34% to 72%. This case describes the novel use of percutaneous needle caecostomy as a life-saving measure to prevent imminent caecal perforation in a 68-year-old lady with large bowel obstruction secondary to an incarcerated incisional hernia. After careful review of computed tomography images and measurement of distances from the abdominal wall to the caecum, the patient's caecum was decompressed in the emergency department using a needle under local anaesthetic. The patient subsequently underwent laparoscopic hernia repair and had an uncomplicated recovery. When conducted safely and with precision in an appropriate patient, percutaneous needle caecostomy can provide immediate symptom relief, reduce risk of caecal perforation, and allow a laparoscopic surgical approach.

## 1. Introduction

Caecal perforation is a life-threatening complication of large bowel obstruction with a competent ileocaecal valve. It has reported mortality ranging from 34% to 72% in case series [1]. This case describes the novel use of percutaneous needle caecostomy as a life-saving measure to prevent imminent caecal perforation in a critically ill patient with large bowel obstruction. When conducted safely and with precision, this technique can provide immediate symptom relief, reduce risk of caecal perforation and subsequent feculent peritonitis, and allow a laparoscopic surgical approach.

## 2. Case Presentation

**Figure 1:**
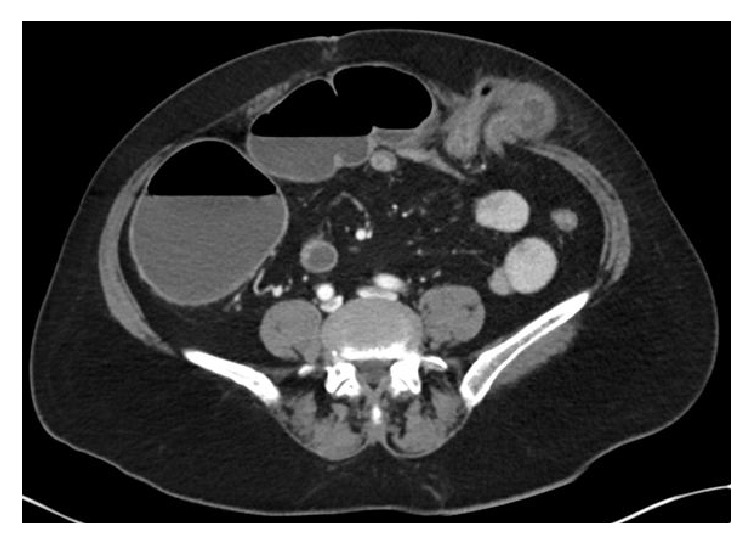
Axial CT showing incisional hernia in the left lower quadrant containing transverse colon causing large bowel obstruction.

**Figure 2:**
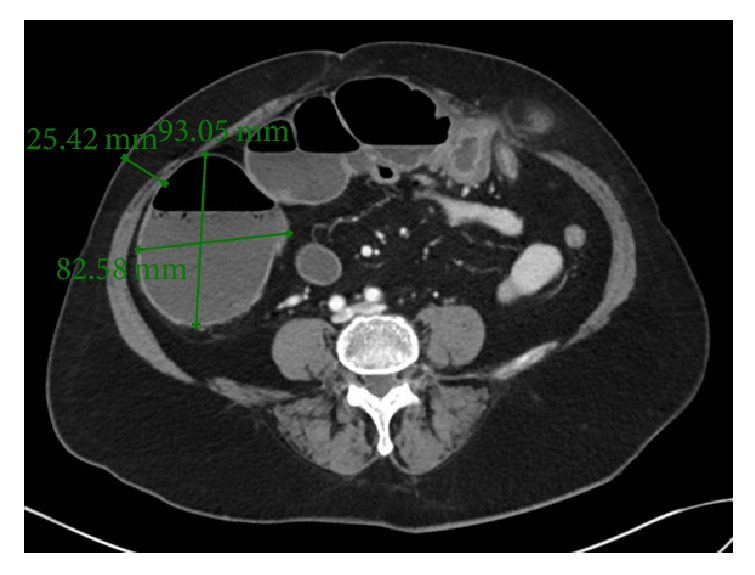
Axial CT showing significantly dilated caecum.

**Figure 3:**
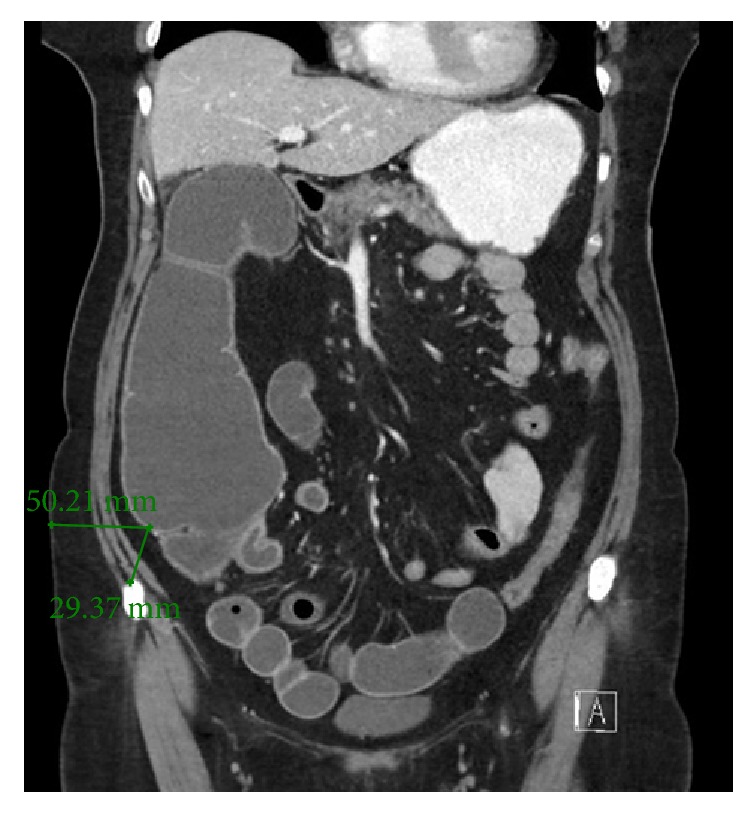
Coronal CT showing caecum measured from the anterior superior iliac spine and the lateral abdominal wall.

**Figure 4:**
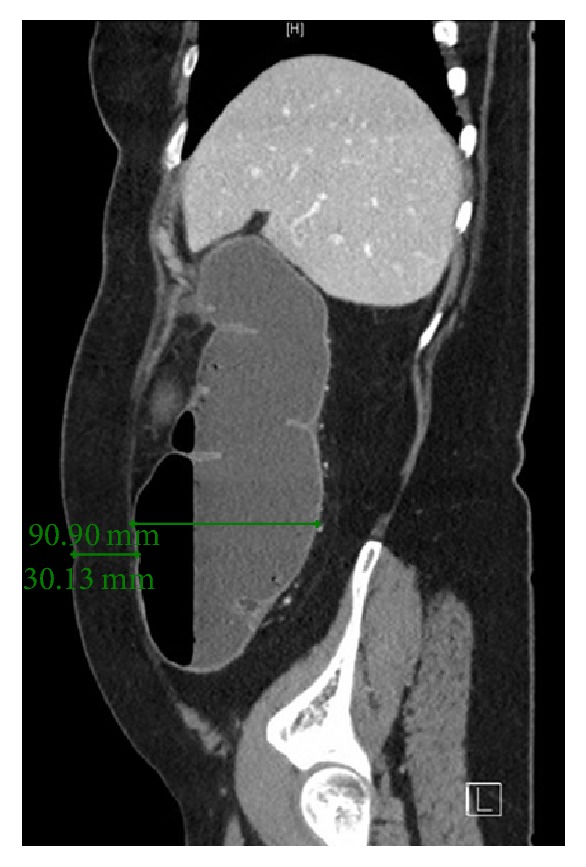
Sagittal CT showing caecum measured from the anterior abdominal wall.

A 68-year-old lady was brought in by ambulance with a one-day history of bilious vomiting, severe colicky abdominal pain, and profuse watery diarrhoea. She had a background of primary lung adenocarcinoma, for which she had completed neoadjuvant chemotherapy 5 months ago. Surgery was abandoned after repeat staging PET/CT scan revealed sacral metastases. Her other medical comorbidities included thalassaemia, osteoporosis, and previous transient ischaemic attack. Her surgical history included an abdominal hysterectomy which was complicated by rectal injury requiring a Hartmann's procedure that was subsequently reversed. Her regular medications were erlotinib 150 mg daily, Asasantin SR, and alendronate. On examination, she had a low grade tachycardia of 90 bpm and blood pressure of 110/70 mmHg. Her abdomen was grossly distended and peritonitic with a palpable hernia containing bowel in the left lower quadrant. Urgent abdominal CT (computed tomography) was performed, which indicated a severe large bowel obstruction secondary to herniation of transverse colon into an incisional hernia at the previous colostomy reversal site (Figure 1). The proximal large bowel was dilated up to 54 mm and the caecum was dilated up to 93 mm in maximum diameter (Figure 2). Distal small bowel loops were also prominent. Given the CT findings of a markedly dilated caecum with clinical features of peritonism the risk of caecal perforation was extremely high. Therefore, an urgent needle decompression of the caecum was performed in the emergency department. Using sagittal and axial CT images, the vertical distance from the anterior superior iliac spine (ASIS) and the anterior abdominal wall skin to the caecum was measured (Figure 3). At the level of the ASIS, the caecum was measured to be approximately 3 cm from the skin and there were no small bowel loops between it and the abdominal wall (Figure 4). The patient was positioned in the partial left lateral position to utilise gravity to mobilise the small bowel away from the caecum. The needle entry point was measured and marked on the skin. Under aseptic technique and local anaesthetic (1% lignocaine), a 21-gauge needle (0.51 mm inner diameter) attached to a 5 ml syringe was inserted through the abdominal wall into the caecum. Correct placement was confirmed by the syringe filling with gas and a small amount of feculent fluid. The plunger of the syringe was then removed and the syringe was attached to low wall suction. This produced effective suction of gas and decompression with immediate improvement in pain and patient comfort. The patient remained haemodynamically stable and subsequently underwent laparoscopic adhesiolysis; reduction of the herniating bowel, mesentery, and omentum; and mesh repair of the abdominal wall defect. At laparoscopy, the caecum was decompressed and appeared pink and viable. There was no peritoneal contamination. As a small (21 G) needle was used for decompression, repair of the temporary caecostomy site was not required. The patient was monitored in the intensive care unit for one day postoperatively before being transferred to the ward, where she had an uncomplicated recovery.

## 3. Discussion

Indications for caecostomy include palliation of malignant bowel obstruction, decompression in chronic intestinal pseudo-obstruction, and administration of anterograde continence enemas in patients with defecation disorders, particularly in patients with spinal cord injury or severe disability [2–4]. Traditional approaches are endoscopic or image-guided percutaneous caecostomy. Endoscopic caecostomy is minimally invasive and has several advantages including direct visualisation (which minimises risk of tube misplacement), the patient is not exposed to ionising radiation, and sedation may be used (which may be preferable to general anaesthetic in patients with significant comorbidities and high surgical risk) [4]. Percutaneous caecostomy is typically performed under CT, fluoroscopic, or less commonly ultrasound guidance [5, 6]. A Seldinger technique is generally used with caecostomy catheter sizes ranging from 10 to 20 French [6]. To our knowledge, no case reports of emergency percutaneous needle decompression of the caecum have been described. Caecum diameter greater than 9 cm is associated with high risk of perforation and mandates urgent intervention [7]. This case demonstrates that, with careful review of CT images, needle caecostomy can be safely performed in select patients as a stabilising measure to reduce risk of caecal perforation and buy time to definitive surgery. Needle caecostomy avoids the need for radiological services, which are in high demand in most hospitals and could delay relief of dilatation. The use of a needle for decompression is less painful than catheter insertion and can be performed under local anaesthetic without the need for sedation. In this case, bowel decompression also allowed laparoscopic hernia repair to be safely performed, thus avoiding the increased postoperative pain and higher risk of complications and morbidities associated with a laparotomy.

There are limitations to the technique of needle caecostomy. It is highly operator dependent and should only be performed after careful review of CT images by a surgical trainee or a surgeon with appropriate experience, followed by radiological or surgical confirmation of technique success. Percutaneous needle caecostomy would also prove difficult in patients with significant body habitus or extensive previous intra-abdominal surgery (due to likely adhesions).

This case shows that needle caecostomy can be effectively and safely performed to produce emergent caecal decompression. However this technique is not advocated for all patients and is not a definitive intervention. It is intended as a life-saving measure in patients with impending caecal perforation.
